# Neuroimaging Applications for Diagnosis and Therapy of Pathologies in the Central and Peripheral Nervous System

**DOI:** 10.3390/brainsci12020207

**Published:** 2022-02-01

**Authors:** Silvia Middei

**Affiliations:** 1 Institute of Biochemistry and Cell Biology, National Research Council, Via E. Ramarini 32, 00015 Monterotondo, Italy; silvia.middei@cnr.it; 2 European Brain Research Institute, Viale Regina Elena 295, 00161 Rome, Italy

Imaging in neurosciences allows for the visual representation of micro- and macro-components of the central (CNS) and peripheral (PNS) nervous systems with the intent of investigating their morphology and function, to provide diagnosis and prognosis of neurological diseases and to monitor responses to treatments. It includes a large variety of in vivo techniques such as the standard Computed Tomography (CT), Magnetic Resonance Imaging (MRI) and Positron Emission Tomography (PET), as well as ex vivo microscopic visualization of single-neuron morphology or gene/protein expression in nervous system tissues. Over the last few years, neuroimaging applications have improved rapidly in order to answer important scientific questions concerning deeper neuroanatomical investigation, the link between genetic alterations and pathological outcomes and the introduction of both new diagnostic biomarkers and tools to ameliorate clinical care.

This Special Issue contains four original research articles, one case report and one review article that answer the above scientific questions through studies that have as common denominator the application of neuroimaging in portions of CNS or PNS. Of these six papers, three focus on human studies and three on animal studies. All of them share an anatomo-clinical approach targeting the following pathologies: Amyotrophic Lateral Sclerosis (ALS), Alzheimer’s Disease (AD), brachial plexus (BP) injury, intracranial carotid artery aneurysm and pontocerebellar hypoplasia (PHC). The imaging techniques applied in the studies include MRI, Flow-diverted stent, immunofluorescence on ex vivo biological samples and micro-CT X-ray imaging. Two papers on human studies involve MRI on the brain of ALS patients and on the brachial plexus of subjects with injury of this group of peripheral nerves. The third paper on human study is a case report using Flow-diverted stent to treat intracranial carotid artery aneurysm. Two of the papers involving animal studies utilize immunofluorescence staining combined with confocal microscopy: The first visualizes microglia markers in the motor cortex of an ALS mouse model, and the second targets somatic peripheral nervous system innervation of skeletal muscle in a mouse model for AD. The third animal study took the opportunity of micro-CT X-ray imaging to assess the expression in intact murine brains of a specific gene involved in PHC. This editorial aims at providing an overview of these six papers and to highlight what the neuroimaging application used in each study adds to the current knowledge in the specific field.

Amyotrophic Lateral Sclerosis (ALS) is a progressive neurodegenerative disease that damages motor neurons in the brain (upper or cortico-spinal motor neurons, CSN) and spinal cord (lower or spinal motor neurons, SMN), causing severe loss of muscle control and gradually leading to complete paralysis and death. In addition to motor neurons, white matter (WM) areas in the ALS brain can also be damaged. In their study, De Marchi et al. [1] aimed at investigating the correlation between the state of WM in both brain and cortico-spinal tract (CST) of ALS patients with their clinical outcomes and their genetic components. To this aim, the authors selected a group of patients clinically diagnosed for ALS, on whom they performed the following: 1. neuropsychological assessment through ALSFRS-R, a functional rating scale that evaluates daily living and global function and typically identifies a linear decline with the disease progression; 2. screening of principal genes associated with ALS including SOD1, C9Orf72, FUS and TARBP; 3. Diffusion Tensor Imaging (DTI) analysis, an MRI-based approach that provides a measure of the microstructural integrity of WM fibre tracts. A few neuroimaging studies using DTI had previously reported both motor and extra-motor WM changes in ALS patients [2,3]. Two DTI measures for WM integrity include fractional anisotropy (FA) and apparent diffusion coefficient (ADC), with decreased FA and increased ADC values, indicating the damage to the axons and myeline of WM fibres. De Marchi and colleagues reported that FA was significantly reduced in Corpus Callosum (CC), Corona Radiata (CR), Cerebral Peduncle (CP), Cerebellar Peduncle (CbP) and CST at posterior limb of internal capsule (CS), while high ADC values were reported in CC and the bilateral CP of ALS patients. In particular, the authors reported a positive correlation between FA values at the right CP and CbP and ALSFRS-R score, indicating that the modest alteration of WM in these two regions is predictive of better clinical diagnosis. No correlation was found between the ADC values and the ALSFRS-R score nor between the FA/ADC values with the expression of genetic mutations in patients. Hence, evidence in this study validates the DTI FA index as an optimal biomarker for ALS clinical diagnosis.

Brachial plexus (BP) is a network of peripheral nerves, formed by the anterior rami of cervical nerves C5-C8 and the first thoracic nerve T1. BP provides sensory and motor innervation to the upper chest, upper limbs and shoulders. Dorsal root ganglion (DRG) is a group of neuronal cell bodies proximal to the convergence of the C5-C8 and T1 spinal nerves. Injuries of the BP are commonly classified as pre-ganglionic if the lesion is proximal to DRG and post-ganglionic if the lesion is distal from it. Diagnosis of BP injury is essential as pre-ganglionic lesions are irreparable while post-ganglionic ones can be treated with surgery if an early diagnosis is achieved. Several diagnostic tools for BP injury are currently available, with MRI considered one of the best for the assessment of both peripheral nerve pathology and adjacent soft tissue and musculature [4]. In a review study, Leigheb et al. [5] provided evidence to further support the use of MRI for pre-surgical diagnosis of BP injury. Through a meta-analysis on a set of recent studies combining MRI with intraoperative findings, Leigheb and colleagues evaluated the diagnostic accuracy of MRI for post-ganglionic lesions. Their analysis indicates a 90% pooled sensitivity of the MRI in detecting post-ganglionic lesions of BP. Furthermore, the study proved that diffusion-weighted imaging with background signal suppression (DWIBS), a method that uses the diffusion of water molecules to generate contrast in MR images, provides an optimal contrast differentiation between nerve fibres and surrounding tissues and strongly contributes to MRI sensitivity. Overall, the study from Leigheb et al. demonstrates the diagnostic value of MRI neuroimaging application for BP injuries.

Intracranial carotid artery aneurysm is a common disease affecting the aged population. Although it is mainly asymptomatic, it is associated with high risk for disability or death in case of rupture. Over the last 20 years, endovascular treatments including coiling have replaced clipping surgery; however, coiling has limitations in the case of wide neck or fusiform aneurysms. An innovative alternative to coiling consists of flow-diverted stent (FDS), an endovascular surgery technique that diverts blow flow away from the aneurism rather than treating the aneurysm itself. The basic principle of FDS consists in disrupting blood flow in proximity of the aneurysm neck but preserving it in parent vessels. Guzzardi et al. [6] report the case study of an 88-year-old woman with intracranial aneurysm and complications due to both tortuosity of the vessel and antiplatelet therapy, which increased the risk of bleeding and prevented the standard femoral artery access for coiling. Authors performed a direct puncture of the common carotid artery with an ultrasound guide, obtaining the complete exclusion of the aneurysm. This clinical study confirms and encourages the use of FDS as a valid alternative to the coiling approach.

In ALS, motor neuron degeneration is accompanied with astrogliosis and microgliosis, but not much is currently known about the involvement of non-neuronal glial cells in the progression of the pathology. Migliarini et al. [7] approached this issue in hSOD^G93A^ mice, which overexpress the G93A mutated form of the human copper-zinc superoxide dismutase (SOD1) gene found in 20% of ALS patients [8]. These mice replicate an age-dependent motor neuron progressive degeneration found in ALS patients and are widely used for studies in ALS. By analysing microglia activation in the motor cortex of early symptomatic hSOD^G93A^ mice, Migliarini et al. aimed at providing a tool for timing microgliosis during ASL progression. To labelled microglia in upper motor neurons, the authors immunostained brain slices, including the motor cortex, with two microglial markers: ionized calcium-binding adaptor molecule 1 (Iba1) and transmembrane protein 119 (TMEM119). Confocal detection of Iba1/TMEM119 co-immunofluorescence was used to identify microglia cells, which were then investigated for their active state by morphological Sholl analysis. This analysis technique allows to assess the number and complexity of ramifications emerging from the microglia cell soma. In fact, microglia exist in distinct states, which are characterized by different morphologies; while normal surveilling microglia have ramified and fine processes, activated microglia display shorter and thicker processes. The study reported a reduced ramification of microglia Iba1/TMEM119-positive cells in the motor cortex of hSOD^G93A^ mice as compared to wild-type controls. This result indicates that microgliosis occurs in the motor cortex of ALS mice in concomitance with the onset of clinical symptoms. This result makes the confocal analysis of the microglia’s state a suitable marker for the identification and characterization for the ALS progression stage.

Alzheimer’s Disease (AD) is the most common form of neurodegenerative disease associated with ageing. Clinical AD signs include cognitive decline and memory loss, but some AD patients also manifest sarcopenia, which is the loss of muscle mass and strength. Acetylcholine (ACh) is a key neurotransmitter expressed in both central and peripheral nervous system, where it regulates cognitive and muscle function, respectively. In AD patients, cognitive decline is strongly associated with the loss of cholinergic neurons, which in turn depends on the presence of amyloid β aggregates, a typical hallmark of AD pathology. Amyloid β is also present in peripheral tissues including the skeletal muscle, where it interferes with ACh release and cholinergic function. Based on these evidences, Torcinaro et al. [9] investigated whether the loss of cholinergic neurons is also evident in skeletal muscles and possibly underlies sarcopenia symptoms in AD. To this, the authors visualised the cholinergic innervation of skeletal muscle tibialis anterior in the Tg2576 mouse model for AD [10]. These mice overexpress amyloid β and manifest sarcopenia symptoms at early stages of AD progression [11]. By means of confocal detection of co-immunofluorescent labelling of Neurofilament Light Chain (NFL) and Choline acetyltransferase (ChAT) which mark neuronal axons and cholinergic neurons, respectively, the authors demonstrated that AD mice display reduced neuritic length and narrower cholinergic synaptic terminals as compared to wild-type controls. These data, which were supported by the reduced expression of cholinergic receptors in skeletal muscle, demonstrate for the first time that cholinergic loss is also evident in the skeletal muscle of AD subjects. Hence, the confocal imaging application used in this study provides a valid tool for the analysis of sarcopenia biomarkers in AD mouse models. Furthermore, this application could eventually be useful for assessing the effects of the pharmacological modulation of the cholinergic system in the cure of sarcopenia in AD and other age-related pathologies.

Pontocerebellar hypoplasia (PCH) is a group of neurodegenerative conditions with genetic origin that alter the development of the brainstem and cerebellum. Other brain regions can also be affected by the pathology, leading to a variety of symptoms that may include microcephaly, developmental delay, movement disorders and intellectual disability. PCH results from mutation in several genes, including the *Tsen54* gene. In their research study, Ermakova et al. [12] aimed to map the expression of the *Tsen54* gene in the brain of healthy mice in order to identify the brain regions more vulnerable to PCH and possibly explain the variability of symptoms. Micro-computed tomography (micro-CT) uses X-ray cross-sections of a physical object to recreate a 3D virtual model. Ermakova et al. applied 3D X-ray imaging for the *LacZ* (β-galactosidase) reporter gene expression analysis in murine brain ex vivo. They demonstrated that the biochemical product of the β-galactosidase reaction enhances the X-ray detectable signal at the sites of the reporter gene activity. They then applied 3D X-ray imaging approach to study tRNA endonuclease 54 (*Tsen54)* gene in intact murine brain. The performed semi-quantitative analysis provided a map of *Tsen54-LacZ* expression in the brain, confirming the hypothesis that the *Tsen54* gene is highly expressed not only in pons and cerebellum but also in brain structures including the isocortex, hippocampal formation, olfactory area, striatum, hypotalamus and medulla that exert a key role in motor and cognitive functions. Hence, this neuroimaging approach is a valid tool for assessing gene expression in intact brains of murine mouse models and has potential implications for the validation of gene-based therapies in CNS pathologies with genetic origin.

Taken together, the papers included in this Special Issue integrate human and animal studies involving neuroimaging applications for diagnosis, prognosis and clinical care of pathologies of the central and peripheral nervous system. One set of these studies provides biomarkers that are relevant for early and accurate diagnosis of nervous system pathologies, with potential application in follow-up studies associated with therapeutic interventions. These biomarkers have been identified at neurobiological, morphological, physiological and genetic levels, providing a huge and multi-level array of diagnostic probes. A second set of studies explores innovative applications of imaging tools for clinical interventions. The main picture emerging from this Special Issue is that neuroimaging provides a set of powerful tools at the crossroad of clinical diagnosis and therapeutic intervention. One may ask whether this potential is fully exploited in a synergistic collaboration between distinct disciplinary areas including radiology, imaging, clinic, neurobiology and genetic. Improved communication among these disciplines, together with the integration of neuroimaging tools with clinical data, may implement best practice guidelines to benefit from available devices for clinical care.
